# Diagnostic Utility of Non-contrast Thoracic Computed Tomography in the Detection of Anemia and Its Severity: A Cross-Sectional Analytical Study

**DOI:** 10.7759/cureus.89901

**Published:** 2025-08-12

**Authors:** Ulaganeyan Damodaran Kumar, Nivya Mary Arputham, S Jaiganesh Sivalingam, Sundararajan Srinivasan

**Affiliations:** 1 Department of Radiology, Meenakshi Medical College Hospital and Research Institute, Meenakshi Academy of Higher Education and Research (Deemed to be University), Kanchipuram, IND

**Keywords:** anemia, blood attenuation, diagnostic accuracy, hounsfield units, thoracic imaging, unenhanced ct

## Abstract

Background

Anemia is a widespread clinical condition that detrimentally influences both quality of life and survival outcomes, particularly in elderly and chronically ill patients. Recent evidence highlights the potential of unenhanced thoracic computed tomography (CT) scans as a non-invasive modality for detecting anemia by assessing blood attenuation values. Our study aims to determine the diagnostic accuracy of unenhanced thoracic CT scans in predicting anemia.

Methods

This was a prospective, single-center, hospital-based analytical cross-sectional study conducted in the Department of Radiology at Meenakshi Medical College Hospital and Research Institute, Kanchipuram District, Tamil Nadu, India, between June 2024 and December 2024.

Results

Among the 225 patients included in the study, 125 (55.6%) were found to have anemia, classified as mild (22.7%), moderate (25.3%), or severe (7.6%). Anemic individuals were significantly older than their non-anemic counterparts (mean age: 59.3 vs. 52.5 years; p = 0.005), though gender distribution showed no significant difference (p = 0.179). Blood attenuation measured in Hounsfield units (HU) on unenhanced thoracic CT was significantly reduced in anemic patients across all vascular sites assessed, including the aortic arch (+33.4 vs. +45.2 HU), ascending aorta (+35.2 vs. +45.1 HU), descending aorta (+36.4 vs. +44.2 HU), and pulmonary artery (+36.3 vs. +45.0 HU), all with p-values < 0.001. Subjective CT signs were also more frequent in the anemic group, with the aortic ring sign present in 42.4% compared to 11.0% in non-anemic patients, and the interventricular septum sign observed exclusively among anemic individuals (16.8%). Hemoglobin levels correlated strongly with HU values across vascular sites, most notably at the aortic arch (r = 0.887), with similarly high correlations in the ascending aorta (r = 0.835) and pulmonary artery (r = 0.787). Receiver operating characteristic (ROC) analysis confirmed excellent diagnostic accuracy, with areas under the curve (AUCs) of 0.953 for anemia at the aortic arch and 0.944 for severe anemia at the pulmonary artery, supporting thoracic CT as a valuable non-invasive tool for anemia detection.

Conclusion

Unenhanced thoracic CT scans demonstrate high diagnostic accuracy in predicting anemia through both objective blood attenuation values and subjective imaging signs. These findings support their use as a rapid, non-invasive adjunct in clinical anemia assessment.

## Introduction

Anemia, defined as a reduced concentration of hemoglobin in the blood, remains a global public health concern affecting approximately one-quarter of the world’s population, with particularly high prevalence in low- and middle-income countries [1,2]. It contributes significantly to morbidity and mortality, especially among vulnerable populations such as the elderly, chronically ill, and individuals with malignancies or chronic inflammatory conditions [3]. Clinical detection of anemia traditionally relies on laboratory-based complete blood count (CBC) testing, which remains the gold standard. However, in many clinical scenarios, particularly in emergency settings, resource-limited areas, or during radiological evaluations for unrelated conditions, where laboratory data may be delayed, unavailable, or overlooked.

Computed tomography (CT) is a medical imaging modality that provides accurate images of the structures within the body from sequential X-ray images. CT can proficiently detect various abnormalities in many different internal organs, soft tissues, bones, and blood vessels within a short span. These privileges have led to the widespread implementation of this modality as a diagnostic tool. With the increasing utilization of thoracic CT for a wide array of diagnostic indications, incidental detection of anemia on non-contrast CT scans has gained interest as a potential supplementary tool for rapid clinical assessment. Unenhanced CT scans inherently visualize blood pool attenuation, measured in Hounsfield units (HU), which is directly influenced by hemoglobin concentration and hematocrit levels [4]. Several studies have revealed that in anemic individuals, the blood in the aorta, pulmonary artery, and other vessels appears hypodense (low intravascular HU) due to lower hemoglobin concentration, thus suggesting a possible role for CT imaging in the indirect diagnosis of anemia [5-7]. Furthermore, qualitative signs such as the “aortic ring sign” and “interventricular septum sign” have been described as visual markers of anemia on CT, though their diagnostic reliability remains subject to radiologist interpretation [8,9].

## Materials and methods

This was a prospective, single-center, hospital-based analytical cross-sectional study conducted in the Department of Radiodiagnosis at Meenakshi Medical College Hospital and Research Institute, Kanchipuram District, Tamil Nadu, India, between June 2024 and December 2024. Institutional Ethics Committee approval was obtained before the commencement of the study, and written informed consent was obtained from all participants after providing a participant information sheet detailing the nature and purpose of the research. Patients were enrolled based on predefined inclusion and exclusion criteria through a complete enumeration of eligible individuals. Consecutive patients aged 15 years and above who underwent unenhanced thoracic CT for non-traumatic medical indications, such as dyspnea, suspected interstitial lung disease, or metastatic workup, etc., were considered for inclusion. Patients in this study were enrolled based on predefined inclusion and exclusion criteria, with complete enumeration of consecutive eligible patients. All patients who consented and met the eligibility criteria and underwent unenhanced thoracic CT for non-traumatic medical indications between June 2024 and December 2024 were included. Though not a complete random sample, there is no section bias in the patients included via complete enumeration, which impacts the interpretation of results. Selecting a random sample for such a study would require a larger sample of patients to be consented and reviewed for eligibility criteria. The results from this study are considered exploratory, and limitations of the study are included in the discussion.

Eligibility criteria required a complete blood count performed within 24 hours of the CT scan. Patients with known histories of iron overload, glycogen storage disorders, recent intravenous contrast administration, ongoing or recent blood transfusions, active bleeding at the time of CT scan, and metal artifacts due to any chest/cardiac implants were excluded.

The sample size was determined based on a reported correlation coefficient of 0.78 between blood attenuation on unenhanced thoracic CT and hemoglobin levels, as shown by Abbasi et al. To detect a similar correlation with 95% confidence and 90% power, the calculated sample size was 213. After adjusting for a 5% non-response rate, the final required sample size was 225 participants. All CT scans were performed using a Toshiba Aquilion Prime 160 Slice CT scanner with acquisition parameters set at 180-450 mAs, 120 kV, and 1 mm section thickness. Images were reconstructed using a soft tissue window, and the radiologist was oblivious to clinical and laboratory results to reduce any bias in the study. Later hemoglobin levels for all the patients were collected and correlated with the imaging findings. 

Subjective assessment included evaluation for the presence of the aortic ring sign (presence of hyperattenuating aortic wall against relatively hypodense blood pool) and interventricular septum sign (hyperattenuating interventricular septum against relatively hypodense blood pool). Objective analysis involved placing circular regions of interest (ROIs) of approximately 1 cm² in standardized anatomical sites, including the ascending and descending thoracic aorta, aortic arch, and main pulmonary artery.

To assess HU in the ascending and descending aorta, the ROI should be placed in the center of the lumens at the level of the T5 vertebra, where both ascending and descending thoracic aorta are visualized. For the aortic arch, the ROI should be placed in the center of the aortic arch lumen, at the level of the tracheal bifurcation, and should avoid the vessel walls to avoid artifacts. For the pulmonary artery, the ROI should be placed in the main pulmonary artery ~1cm above the bifurcation. This specific localization of ROI allows for consistent and reliable measurements of blood density, which is correlated with hemoglobin levels and anemia severity.

The interventricular septum was excluded from ROI-based objective analysis due to its variable visibility on non-contrast studies. ROIs were carefully placed to avoid artifacts and ensure consistency. The mean HU values from each ROI were recorded using a dedicated workstation. These findings are subjective and reader-dependent and subject to interobserver variability.

Hemoglobin values were classified according to the World Health Organization criteria: for non-pregnant women aged 15 years and above, non-anemia was defined as hemoglobin ≥12.0 g/dL, mild anemia as 11.0-11.9 g/dL, moderate anemia as 8.0-10.9 g/dL, and severe anemia as <8.0 g/dL; for men aged 15 years and above, non-anemia was defined as hemoglobin ≥13.0 g/dL, mild anemia as 11.0-12.9 g/dL, moderate anemia as 8.0-10.9 g/dL, and severe anemia as <8.0 g/dL [10].

Statistical analysis

Data were entered into MS Excel (Microsoft Corporation, Redmond, Washington, United States) and analyzed using IBM SPSS Statistics for Windows, Version 26 (Released 2018; IBM Corp., Armonk, New York, United States). Continuous variables were summarized using mean and standard deviation (SD), while categorical variables were expressed as frequencies and percentages. The normality of distribution for continuous variables was assessed using the Shapiro-Wilk test as a pooled data. Group comparisons between anemic and non-anemic participants were performed using the independent samples t-test for normally distributed variables and the chi-square test for categorical variables. Pearson’s correlation coefficient (r) was used to assess the strength and direction of linear relationships between hemoglobin levels and mean blood attenuation (HU) at various thoracic vascular sites. Receiver operating characteristic (ROC) curve analysis was employed to evaluate the diagnostic accuracy of blood attenuation in predicting anemia and severe anemia. The area under the curve (AUC), optimal cut-off values, sensitivity, specificity, and 95% confidence intervals (CIs) were reported for each vascular region. A p-value of <0.05 was considered statistically significant. The results from all patients included in the study are summarized. There was no validation set used to confirm the finding in an independent sample. The results from this study are considered exploratory, and limitations of the study are included in the discussion.

## Results

Of the 225 patients included in the study, a total of 125 patients (55.6%) had anemia: 51 (22.7%) had mild, 57 (25.3%) had moderate, and 17 patients (7.6%) had severe anemia (Table 1). Patients with anemia were significantly older than their non-anemic counterparts, with a mean age of 59.3 ± 15.9 years compared to 52.5 ± 19.7 years (p = 0.005). There was no statistically significant difference in gender distribution between the two groups (p = 0.179) (Table 2). Mean hemoglobin levels were significantly lower in the anemic group (10.2 ± 1.9 g/dL) compared to the non-anemic group (13.7 ± 1.3 g/dL; p < 0.001).

**Table 1:** Comparative frequency of subjective CT signs (aortic ring and interventricular septum) in anemic vs. non-anemic patients (n = 225) with Chi-square (χ²) test results χ² = Chi-square; p < 0.05 considered statistically significant

CT sign	Group	Sign present, N (%)	Sign absent, N (%)	Total (n)	χ² value	p-value
Aortic ring sign	Anemic	53 (42.4%)	72 (57.6%)	125	29.28	<0.001
	Non-anemic	11 (11.0%)	89 (89.0%)	100		
Interventricular septum sign	Anemic	21 (16.8%)	104 (83.2%)	125	20.19	<0.001
	Non-anemic	0 (0.0%)	100 (100%)	100		

**Table 2:** Comparison of anemic and non-anemic patients by demographic characteristics, hemoglobin, mean blood attenuation in various anatomic locations, presence/absence of aortic arch, interventricular septum SD: standard deviation

		Anemic N = 125	Non-anemic N = 100	Chi-square/t value (p-value)
Age (in years), mean (SD)		59.3 (15.9)	52.5 (19.7)	2.865 (0.005*)
Gender, n (%)	Female	65 (52.0)	43 (43.0)	1.460 (0.179)
	Male	60 (48.0)	57 (57.0)	1.460 (0.179)
Hemoglobin (in gm/dl), mean (SD)		10.2 (1.9)	13.7 (1.3)	-15.710 (<0.001*)
Hounsfield unit				
Aortic arch, mean (SD)		33.4 (4.3)	45.2 (5.5)	-18.062 (<0.001*)
Ascending aorta, mean (SD)		35.2 (5.2)	45.1 (5.5)	-13.831 (<0.001*)
Descending aorta, mean (SD)		36.4 (5.5)	44.2 (4.5)	-11.444 (<0.001*)
Pulmonary artery, mean (SD)		36.3 (5.5)	45.0 (5.4)	-11.886 (<0.001*)
Other CT assessments				
Aortic arch, n (%)	Present	53 (42.4)	11 (11.0)	25.391 (<0.001*)
	Absent	72 (57.6)	89 (89.0)	25.391 (<0.001*)
Interventricular septum, n (%)	Present	21 (16.8)	0 (0.0)	16.597 (<0.001*)
	Absent	104 (83.2)	100 (100)	16.597 (<0.001*)
*statistically significant at p < 0.05				

**Figure 1:**
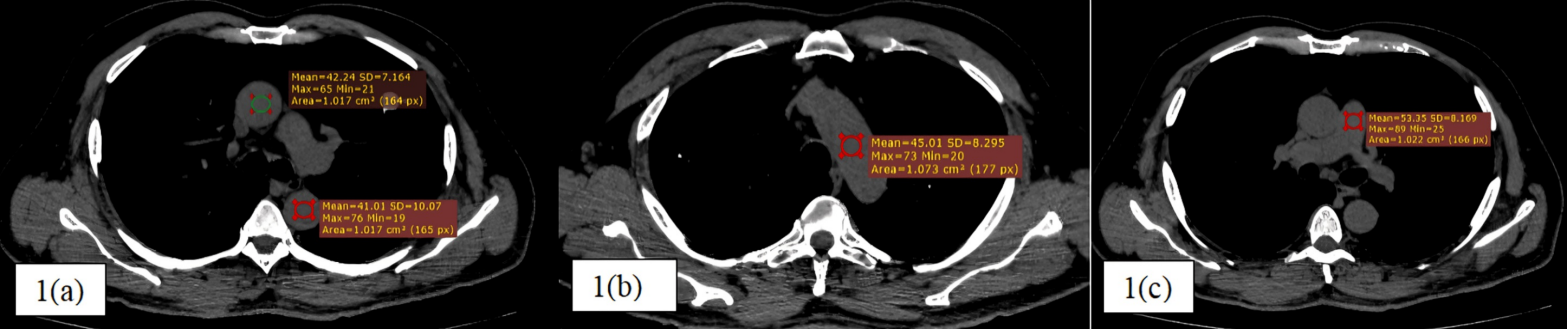
Non-contrast CT thorax-mediastinal window of a 56-year-old non-anemic male patient (serum hemoglobin 15.2 g/dl) showing the circular regions of interest (ROIs) of approximately 1 cm² in standardized anatomical sites, the ascending and descending thoracic aorta (a), aortic arch (b), and main pulmonary artery (c) with mean HU as +42.2, +41.0, +45.0, and +53.3, respectively, which falls within normal limits of non-anemic patients

**Figure 2:**
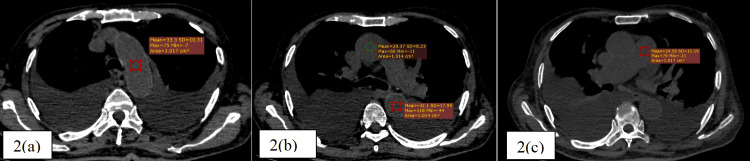
Non-contrast CT thorax-mediastinal window of a 74-year-old severe anemic male patient (serum hemoglobin 7.2 g/dl) showing the circular regions of interest (ROIs) in standardized anatomical sites, the ascending and descending thoracic aorta (a), aortic arch (b), and main pulmonary artery (c) with mean HU as +29.3, +32.1, +33.3, and +24.5, respectively

The mean HU values in all vascular regions assessed on unenhanced thoracic CT were significantly lower in anemic individuals: aortic arch (33.4 ± 4.3 vs. 45.2 ± 5.5), ascending aorta (35.2 ± 5.2 vs. 45.1 ± 5.5), descending aorta (36.4 ± 5.5 vs. 44.2 ± 4.5), and pulmonary artery (36.3 ± 5.5 vs. 45.0 ± 5.4), all with p-values < 0.001 in comparison to non-anemic individuals (Figures 1, 2).

**Figure 3:**
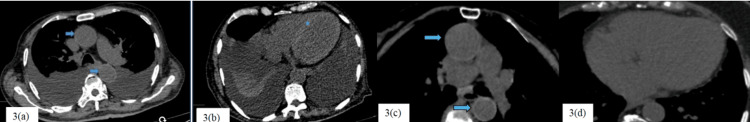
(a, b) Non-contrast CT thorax of a 74-year-old patient with severe anemia showing aortic ring sign (arrow in a) and interventricular septum sign (star in b). (c, d) Non-contrast CT thorax of a 56-year-old patient with mild anemia showing aortic ring sign (arrow in c); however, there is no interventricular septal sign seen (d)

Additionally, subjective CT signs such as the aortic ring sign and interventricular septum sign were significantly more frequent in anemic patients, with the aortic ring sign present in 42.4% of anemic cases compared to 11.0% of non-anemic individuals (p < 0.001), and the interventricular septum sign observed exclusively among anemic patients (16.8%; p < 0.001) (Figure 3).

**Figure 4:**
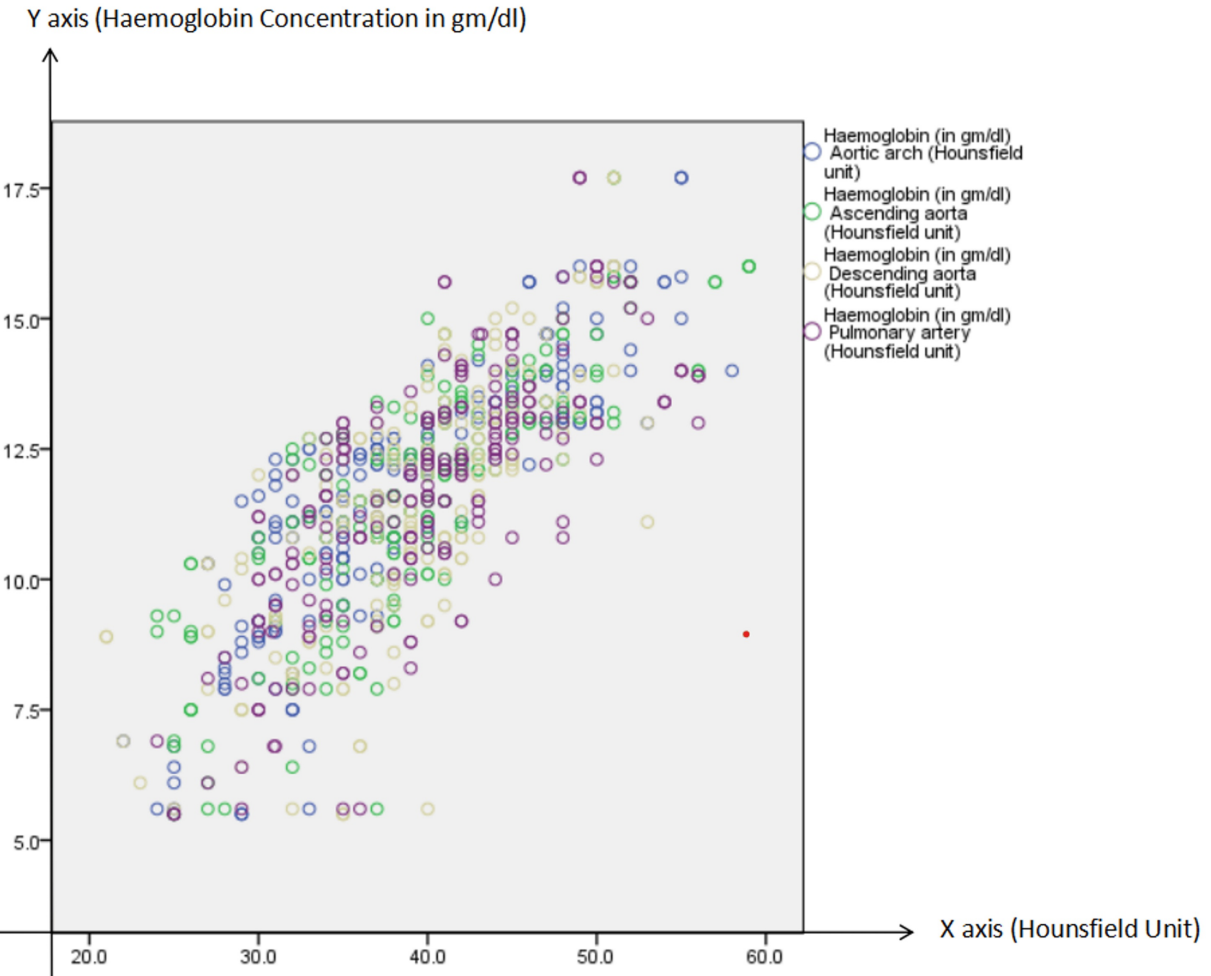
Correlation between hemoglobin and mean blood attenuation in various anatomic locations (overall)

There was a strong and statistically significant positive correlation between hemoglobin levels and mean blood attenuation (measured in HU) across all evaluated vascular regions on unenhanced thoracic CT scans. Overall, the strongest correlation was observed at the aortic arch (r = 0.887, p < 0.001), followed by the ascending aorta (r = 0.835), pulmonary artery (r = 0.787), and descending aorta (r = 0.766). When stratified by age, individuals younger than 60 years exhibited slightly stronger correlations, particularly at the aortic arch (r = 0.902), compared to those aged 60 years and above (r = 0.842) (Figure 4).

Gender-wise analysis revealed consistently strong correlations in both males and females, with males demonstrating higher correlation coefficients across most regions, most notably in the aortic arch (r = 0.895) and ascending aorta (r = 0.837). All correlations were statistically significant with p-values < 0.001 (Table 3).

**Table 3:** Correlation between hemoglobin and mean blood attenuation in various anatomic locations, by overall, age and gender

	Aortic arch	Ascending aorta	Descending aorta	Pulmonary artery
	r (p-value)	r (p-value)	r (p-value)	r (p-value)
Overall	0.887 (<0.001*)	0.835 (<0.001*)	0.766 (<0.001*)	0.787 (<0.001*)
Age <60	0.902 (<0.001*)	0.829 (<0.001*)	0.776 (<0.001*)	0.759 (<0.001*)
Age >60	0.842 (<0.001*)	0.828 (<0.001*)	0.778 (<0.001*)	0.809 (<0.001*)
Gender, female	0.806 (<0.001*)	0.791 (<0.001*)	0.677 (<0.001*)	0.799 (<0.001*)
Gender, male	0.895 (<0.001*)	0.837 (<0.001*)	0.760 (<0.001*)	0.733 (<0.001*)
r, Pearsons’ correlation coefficient *statistically significant at p < 0.05				

**Figure 5:**
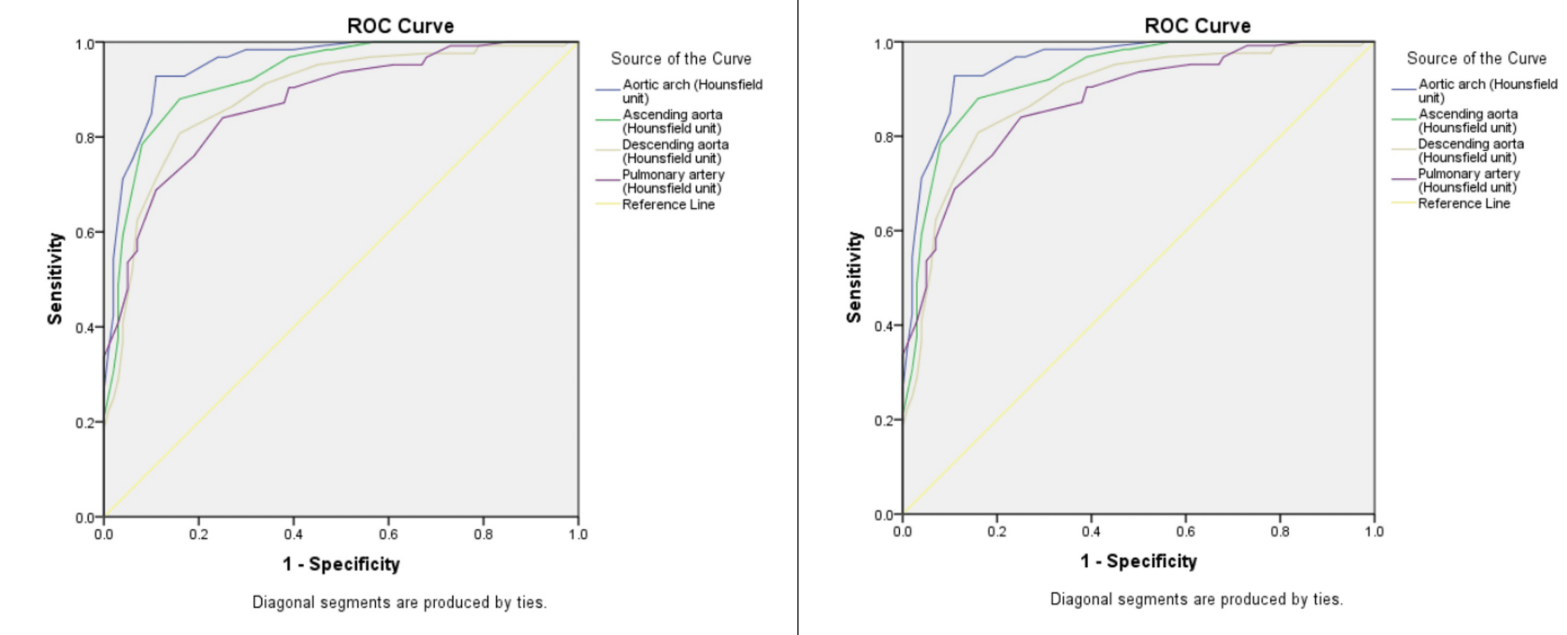
ROC analysis showing the AUC of blood attenuation at aortic arch, ascending aorta, descending aorta, and pulmonary artery to predict presence of anemia and severe anemia AUC: area under the curve​​​​​​; ROC: receiver operating characteristic

ROC analysis demonstrated excellent diagnostic performance of blood attenuation measurements across all evaluated vascular sites for predicting both anemia and severe anemia. The ROC cut-off thresholds were data-derived. The highest AUC for anemia detection was observed at the aortic arch (AUC = 0.953; 95% CI: 0.927-0.979), with a cut-off value of <37 HU yielding 84.8% sensitivity and 90.0% specificity (p < 0.001). For severe anemia, the pulmonary artery showed the highest AUC (0.944; 95% CI: 0.904-0.983), with a cut-off of <35 HU providing 88.2% sensitivity and 81.2% specificity. Blood attenuation at the ascending aorta also demonstrated high diagnostic accuracy for both anemia (AUC = 0.926) and severe anemia (AUC = 0.934), with cut-off values >40 HU and >35 HU, respectively. The descending aorta, while slightly lower in diagnostic performance, still showed robust AUCs of 0.885 for anemia and 0.889 for severe anemia, with good sensitivity and specificity values. All associations were statistically significant (p < 0.001), supporting the utility of thoracic CT blood attenuation as a reliable non-invasive indicator of anemia severity (Table 4, Figure 5).

**Table 4:** ROC analysis showing the AUC of blood attenuation at aortic arch, ascending aorta, descending aorta, and pulmonary artery to predict the presence of anemia and severe anemia AUC: area under the curve​​​​​​; CI: confidence interval; ROC: receiver operating characteristic; SE: standard error. *p < 0.05 denotes statistical significance.​​​​​ Z-statistics reflect the test of significance for AUC values >0.5 using the formula: Z = (AUC – 0.5)/SE

	AUC (95% CI)	Cut off	Sensitivity (95% CI)	Specificity (%)	p-value	Z-statistic
Aortic arch						
Anemia	0.953 (0.927 to 0.979)	>37	84.8 (77.5-90.0)	90.0 (82.6-94.5)	<0.001*	13.49
Severe anemia	0.927 (0.882 to 0.971)	>32	100.0 (81.6-100.0)	77.9 (71.8-83.0)	<0.001*	9.85
Ascending aorta						
Anemia	0.926 (0.891 to 0.960)	>40	88.0 (81.1-92.6)	84.0 (75.6-89.9)	<0.001*	10.74
Severe anemia	0.934 (0.890 to 0.978)	>35	88.2 (65.6-96.7)	83.2 (77.5-87.7)	<0.001*	9.96
Descending aorta						
Anemia	0.885 (0.840 to 0.929)	>39	71.2 (62.7-78.4)	89.0 (81.4-93.7)	<0.001*	9.04
Severe anemia	0.889 (0.831 to 0.948)	>37	94.1 (73.0-98.9)	81.2 (75.3-85.9)	<0.001*	8.11
Pulmonary artery						
Anemia	0.872 (0.828 to 0.917)	>40	76.0 (67.8-82.6)	81.0 (72.2-87.5)	<0.001*	8.51
Severe anemia	0.944 (0.904 to 0.983)	>35	88.2 (65.6-96.7)	81.2 (75.3-85.9)	<0.001*	10.29

## Discussion

This study decisively shows that unenhanced thoracic CT is a valuable non-invasive tool for predicting anemia, with strong correlations observed between hemoglobin levels and blood attenuation (HU) in key thoracic vascular structures. Among the 225 participants, anemia was prevalent in 55.6%, a figure that reflects the global and national burden of anemia, particularly in older and chronically ill populations [11,12]. The significant age difference between anemic and non-anemic individuals in this study (mean age 59.3 vs. 52.5 years) is consistent with existing evidence that advancing age is associated with an increased prevalence of anemia due to factors such as chronic disease, nutritional deficiencies, and bone marrow suppression [13]. Gender distribution did not differ significantly between groups, aligning with prior studies indicating that while anemia thresholds vary by sex, prevalence may not consistently show sex-specific patterns after adjusting for age and clinical comorbidities [14]. The marked reduction in hemoglobin levels in the anemic group vs the non-anemic group(10.2 g/dL vs. 13.7 g/dL) is reflected in a corresponding decline in CT-measured blood attenuation across all evaluated vascular sites. These findings corroborate earlier reports by Abbasi et al., who demonstrated that reduced intravascular HU values on non-contrast CT could reliably indicate anemia, with attenuation values being proportional to circulating hemoglobin concentrations [5].

The aortic arch emerged as the most reliable site for detecting anemia, with significantly lower HU values in anemic patients (33.4 ± 4.3 vs. 45.2 ± 5.5) and a correlation coefficient of r = 0.887 (p < 0.001) between hemoglobin levels and HU attenuation. This finding may be attributed to the relative consistency in anatomic localization and minimal motion artifact in the aortic arch region, making it a suitable site for reproducible measurements [15]. The ascending and descending aorta, along with the pulmonary artery, also demonstrated strong correlations, affirming the robustness of CT-based blood attenuation as a surrogate marker of anemia across diverse anatomical locations. Age-stratified analysis revealed that patients under 60 years exhibited slightly stronger correlations (e.g., aortic arch: r = 0.902) compared to those over 60 years (r = 0.842). These differences may reflect age-related changes in vascular compliance and hematologic profiles that can influence CT attenuation [16]. Gender-based analysis showed higher correlation coefficients in males than females, possibly reflecting less variability in body composition and hematocrit in males, as supported by previous imaging studies examining intravascular density patterns [17].

Subjective signs such as the aortic ring and interventricular septum signs were significantly more frequent among anemic patients (42.4% and 16.8%, respectively), with the interventricular septum sign being exclusively observed in the anemic group. These qualitative findings augment the objective measurements and have been described in earlier radiological literature as visually appreciable signs of low blood attenuation consistent with anemia [18-22]. While these signs may be operator-dependent, their statistically significant association with anemia status in the current study supports their adjunctive diagnostic value.

The present study establishes the diagnostic value of unenhanced thoracic CT in identifying both anemia and severe anemia, as demonstrated by high AUC values across multiple intrathoracic vascular structures in ROC analysis. The aortic arch showed the highest diagnostic accuracy for detecting anemia, with an AUC of 0.953 (95% CI: 0.927-0.979), reflecting excellent discriminatory power. A cut-off attenuation value of <37 HU yielded a sensitivity of 84.8% and a specificity of 90.0%, indicating that attenuation measurements at this location provide a balanced and reliable indicator of anemia status. These findings align with prior research by Abbasi et al., who demonstrated that attenuation values measured on non-contrast chest CT correlated strongly with hemoglobin levels and could serve as a non-invasive marker for anemia. Similar diagnostic efficacy was also observed in the current study for the ascending aorta (AUC = 0.926) and descending aorta (AUC = 0.885), as well as the pulmonary artery (AUC = 0.872), reinforcing the robustness of vascular attenuation as a proxy for blood hemoglobin concentration across various anatomical locations. The pulmonary artery, while less frequently emphasized in previous literature, emerged as the most accurate site for detecting severe anemia, with an AUC of 0.944 (95% CI: 0.904-0.983), a cut-off value of <35 HU, and associated sensitivity and specificity of 88.2% and 81.2%, respectively. This suggests that in severe anemia, attenuation in the pulmonary circulation remains a reliable marker, likely due to its central location and consistent visualization on axial CT slices. These results are supported by data from Abbasi et al., who highlighted the utility of major central vessels, particularly the pulmonary artery and aorta, for estimating blood characteristics non-invasively [5].

The clinical utility of these findings is particularly relevant in emergency and resource-limited settings, where laboratory assessments may be delayed or unavailable. The incorporation of HU-based thresholds into routine interpretation of non-contrast thoracic CT scans could facilitate early detection of anemia, prompting timely hematologic evaluation and intervention. The cut-off values derived from this study (<37 HU for the aortic arch and <35 HU for the pulmonary artery in severe anemia) provide practical benchmarks for clinical application and may guide radiologists in flagging potential cases for further work-up. The ROC cut-off thresholds were purely data-derived as specified in the results section.

The present study has several limitations that warrant consideration. First, as a single-center, hospital-based study, the findings may not be generalizable to broader populations, particularly those outside tertiary care settings or with differing demographic and clinical profiles. Second, although all CT scans were interpreted by an experienced radiologist, the subjective signs, such as the aortic ring and interventricular septum signs, may be prone to interobserver variability, which was not formally assessed in this study. Third, while objective attenuation measurements were standardized using circular regions of interest, variations in image acquisition parameters, patient positioning, and intrinsic factors such as cardiac output or hydration status may have influenced blood attenuation values. Interobserver and intraobserver variability for the ROI were not assessed. Data to assess interobserver and intraobserver variability or correlations for ROI were not collected. Additionally, the study excluded patients with recent blood transfusions, active bleeding, or contrast administration, which may limit applicability in acute care scenarios where such conditions are common. Finally, the cross-sectional design precludes assessment of temporal changes in hemoglobin or attenuation values, and no comparison was made with other imaging modalities or biomarkers for anemia, which could have strengthened the validity of the conclusions.

## Conclusions

In conclusion, this study demonstrates that unenhanced thoracic CT scans provide a reliable and non-invasive method for predicting anemia through both objective attenuation measurements and subjective radiological signs. Significant correlations were observed between hemoglobin levels and mean blood attenuation across major thoracic vascular structures, with the aortic arch and pulmonary artery showing the highest diagnostic accuracy for detecting anemia and severe anemia, respectively. These findings support the integration of attenuation-based assessment into routine CT interpretation, particularly in settings where laboratory data may be delayed or unavailable, thereby enhancing early detection and clinical management of anemia. Thus, an increase in the available modalities, like unenhanced CT, could increase the rate of detection in the early stages and thus prevent further complications while improving patient outcomes.
